# Supplementary material for: Successful promotion of physical activity among students of medicine through motivational interview and Web-based intervention
Source: PeerJ. 2020 Jul 8;8:e9495. doi: 10.7717/peerj.9495 (PMC7353914; doi:10.7717/peerj.9495)
Supplement: Supplemental Information 2 [file peerj-08-9495-s002.pdf]

Annex 1. Questionnaire translated in English

University of Belgrade
Faculty of Medicine

Thank you for agreeing to take part in this important survey.

We will be gaining your thoughts and opinions in order to improve your health and to plan interventions for providing University students the best health care possible.
The main promoters of this research are physicians belonging to the academic staff of the Institute of Social Medicine Faculty of Medicine University of Belgrade.
Be assured that all answers you provide will be kept in the strictest confidentiality.
We ask you to fill in a secret code. Your code is made up of your birth date, the last two letters of your first name and the first two letters of your last name.

For instance:

Mary's day of birth		Mary's month of birth			Last two letters of Mary's name		First two letter of Mary's surname	
1	4	O	C	T	R	Y	R	O

Following the example, create your own secret code:

Your day of birth		Your month of birth			Last two letters of your name		First two letter of your surname	

Copyright: Institut za socijalnu medicinu; Centar škole javnog zdravlja i menadžmenta u sistemu zdravstvene zaštite
2016.

A01. **Gender**: □ Male □ Female

A02. **Age**: _________

A03. **Nationality**: ____________________________

A04. **Mode of transport to School:**
□ Walk □ Bike □ Scooter/motorcycle
□ Car □ Public transportation □ other: ______________________________

A05. **Average mark on university**:

A06. **Commuting time to School** (in minutes)**:_______**

A07. **Height** (in cm, measured today by physician)**:_______**

A08. **Weight** (in kg, measured today by physician)**:_______**

A09: **Waist circumference (in cm) for females: _____**

A10: **Waist circumference (in cm) for males: _____**

A11. **Marital status**:
□ Single (never married) □ In a relationship (including marriage and registered partnership)
□ Widowed □ Divorced

A12. **What is approximately your monthly income?**

□ <100€ □ 100-200€ □ 200-300€ □ 300-400€ □ >400€

A13. **How would you rate the said monthly income?**
□ Very good □ Good □ Fair □ Not good □ Not good at all

A14. **What is approximately the monthly income per capita of your family?**
□ <300€ □ 300-400€ □ 400-500€ □ 500-600€ □ >600€

A15. **How would you rate the said monthly income?**
□ Very good □ Good □ Fair □ Not good □ Not good at all

A16. **How is your health in general?**
□ Very good □ Good □ Fair □ Not good □ Not good at all

Actual level of physical activity

Next questions regard the time you spend doing different types of physical activity **in the last 7 days**. Please note that 'vigorous-intensity activities' are activities that require hard physical effort and cause large increases in

Copyright: Institut za socijalnu medicinu; Centar škole javnog zdravlja i menadžmenta u sistemu zdravstvene zaštite 2016.

breathing or heart rate, while 'moderate-intensity activities' are activities that require moderate physical effort and cause small increases in breathing or heart rate.

B01. How many days did you do vigorous physical activities? __________ **days per week**

B02. How many minutes did you spend on one of those days doing vigorous physical activities? __________ **minutes per day**

B03. How many days did you do moderate physical activities? __________ **days per week**

B04. How many minutes did you spend on one of those days doing moderate physical activities? _________ **minutes per day**

B05. How many days did you walk for at least 10 minutes at a time? __________ **days per week**

B06. How many minutes did you spend on one of those days walking? ___________ **minutes per day**

B07. How many minutes did you spend sitting? ___________ **minutes per day**

Attitude toward level of physical activity

B08. Have you been satisfy with the level of physical activity during previous week?

□ Yes □ No

B09. If not, which sentence describe your reasons?

□ No time □ Too tired □ No wish □ Do not like □ Other

B10. Do you have a wish to be more active in the next 6 months?

□ Yes □ No

Wish for physical activity

Please, do think: what is optimal level of your physical activity aligned with your daily obligations.

B11. How many days would you do vigorous physical activities? __________ **days per week**

B12. How many minutes would you spend on one of those days doing vigorous physical activities? __________ **minutes per day**

B13. How many days would you do moderate physical activities? __________ **days per week**

B14. How many minutes would you spend on one of those days doing moderate physical activities? _________ **minutes per day**

Copyright: Institut za socijalnu medicinu; Centar škole javnog zdravlja i menadžmenta u sistemu zdravstvene zaštite 2016.

B15. How many days would you walk for at least 10 minutes at a time? __________ **days per week**

B16. How many minutes would you spend on one of those days walking? ___________ **minutes per day**

Type of preferred physical activity

B17. Which one is your favorite physical activity?

□ Walk □ Jogging □ Individual jim □ Group jim □ Group sports □ Swimming □ Skiing
□ Dance □ Other:___________

Planned physical activity

B18. In the next 1 months, do you have a plan to practice some od the following physical activities?

Copyright: Institut za socijalnu medicinu; Centar škole javnog zdravlja i menadžmenta u sistemu zdravstvene zaštite 2016.

For each of the following questions in this page, number zero represents the worst and number ten the best your life can be. For every domain, please think about present, past and future.

When it comes to the **best possible life** for you, on which number...

C01.1. ... do you stand **now**?	0	1	2	3	4	5	6	7	8	9	10
C01.2. ... did you stand **a year ago**?	0	1	2	3	4	5	6	7	8	9	10
C01.3. ... do you think you will stand **a year from now**?	0	1	2	3	4	5	6	7	8	9	10

When it comes to **relationships with important people** in your life, on which number...

C02.1. ... do you stand **now**?	0	1	2	3	4	5	6	7	8	9	10
C02.2. ... did you stand **a year ago**?	0	1	2	3	4	5	6	7	8	9	10
C02.3. ... do you think you will stand **a year from now**?	0	1	2	3	4	5	6	7	8	9	10

When it comes to the **community** where you live, on which number...

C03.1. ... do you stand **now**?	0	1	2	3	4	5	6	7	8	9	10
C03.2. ... did you stand **a year ago**?	0	1	2	3	4	5	6	7	8	9	10
C03.3. ... do you think you will stand **a year from now**?	0	1	2	3	4	5	6	7	8	9	10

When it comes to your **student status**, on which number...

C04.1. ... do you stand **now**?	0	1	2	3	4	5	6	7	8	9	10
C04.2. ... did you stand **a year ago**?	0	1	2	3	4	5	6	7	8	9	10
B04.3. ... do you think you will stand **a year from now**?	0	1	2	3	4	5	6	7	8	9	10

When it comes to your **physical health and wellness**, on which number...

C05.1. ... do you stand **now**?	0	1	2	3	4	5	6	7	8	9	10
C05.2. ... did you stand **a year ago**?	0	1	2	3	4	5	6	7	8	9	10
C05.3. ... do you think you will stand **a year from now**?	0	1	2	3	4	5	6	7	8	9	10

Copyright: Institut za socijalnu medicinu; Centar škole javnog zdravlja i menadžmenta u sistemu zdravstvene zaštite 2016.

When it comes to your **emotional and psychological well-being**, on which number...

C06.1. ... do you stand **now**?	0	1	2	3	4	5	6	7	8	9	10
C06.2. ... did you stand **a year ago**?	0	1	2	3	4	5	6	7	8	9	10
C06.3. ... do you think you will stand **a year from now**?	0	1	2	3	4	5	6	7	8	9	10

When it comes to your **economic situation**, on which number...

C07.1. ... do you stand **now**?	0	1	2	3	4	5	6	7	8	9	10
C07.2. ... did you stand **a year ago**?	0	1	2	3	4	5	6	7	8	9	10
C07.3. ... do you think you will stand **a year from now**?	0	1	2	3	4	5	6	7	8	9	10

When it comes to your **sexual life**, on which number...

C08.1. ... do you stand **now**?	0	1	2	3	4	5	6	7	8	9	10
C08.2. ... did you stand **a year ago**?	0	1	2	3	4	5	6	7	8	9	10
C08.3. ... do you think you will stand **a year from now**?	0	1	2	3	4	5	6	7	8	9	10

Over the last 2 weeks, how often have you been bothered by any of the following problems?

	Not at all	Several days	More than half the days	Nearly every day
D01. Little interest or pleasure in doing things	☐	☐	☐	☐
D02. Feeling down, depressed, or hopeless	☐	☐	☐	☐
D03. Trouble falling or staying asleep, or sleeping too much	☐	☐	☐	☐
D04. Feeling tired or having little energy	☐	☐	☐	☐
D05. Poor appetite or overeating	☐	☐	☐	☐
D06. Feeling bad about yourself — or that you are a failure or have let yourself or your family down	☐	☐	☐	☐
D07. Trouble concentrating on things, such as reading the newspaper or watching television	☐	☐	☐	☐
D08. Moving or speaking so slowly that other people could have noticed? Or the opposite — being so fidgety or restless that you have been moving around a lot more than usual	☐	☐	☐	☐

Copyright: Institut za socijalnu medicinu; Centar škole javnog zdravlja i menadžmenta u sistemu zdravstvene zaštite
2016.

The last set of questions regards lifestyle factors

E01. **Have you ever used antihypertensive medicines?**

□ Yes □ No

E02. **Have you ever measured high blood sugar level (during routine check or during pregnancy)?**

□ Yes □ No

E03. **Do you have diabetes in your family?**

□ Yes (parents, brother, sister, child) □ Yes (grandparents, uncle, aunt, first relatives) □ No

E04. **Do you smoke at all nowadays?**

□ Yes, daily □ Yes, occasionally (less than 30 cigarettes in the last month) □ Not at all

E05. **During the past 12 months, have you taken any cannabis?** □ Yes □ No

E06. **During the past 12 months, have you taken any other substance, such as cocaine, amphetamines, ecstasy or other similar substances?** □ Yes □ No

E07. **During the past 12 months, how often did you have 6 or more drinks on one occasion?**

□ Never □ Less than monthly □ Weekly □ Daily or almost daily

E08. **How often do you eat fruits (excluding juice)?**

□ Never □ Less than once a week □ 1-3 times per week

□ 4-6 times per week □ Once a day □ Twice or more a day

E09. **How often do you eat vegetables or salad (excluding juice and potatoes)?**

□ Never □ Less than once a week □ 1-3 times per week

□ 4-6 times per week □ Once a day □ Twice or more a day

Write your email address:____

The survey is complete. Thank you once more for the time you dedicated us. If you want more information or details on this research, please contact your teaching assistant at the Institute of Social Medicine.

The survey is complete. Thank you once more for the time you dedicated us.

Copyright: Institut za socijalnu medicinu; Centar škole javnog zdravlja i menadžmenta u sistemu zdravstvene zaštite 2016.

Copyright: Institut za socijalnu medicinu; Centar škole javnog zdravlja i menadžmenta u sistemu zdravstvene zaštite 2016.
