# Supplementary material for: Successful promotion of physical activity among students of medicine through motivational interview and Web-based intervention
Source: PeerJ. 2020 Jul 8;8:e9495. doi: 10.7717/peerj.9495 (PMC7353914; doi:10.7717/peerj.9495)
Supplement: Supplemental Information 3 [file peerj-08-9495-s003.pdf]

Annex 2. Motivational interview protocol translated in English

University of Belgrade
Faculty of Medicine
Institute of Social Medicine

Dr Subotića 8
11000 Beograd, Srbija
Tel: +381 11 2643 830, Fax: +381 11 2659 533

Protocol for Motivational Interview

Motivational interviewing (MI) is a client-centered, guiding method of communication & counseling to elicit and strengthen motivation for change. The motivational interview involves interviewing four processes: engaging, focusing, evoking, and planning, through which four necessary communication skills of counseling in a motivational interview are imbued. These skills are asking open-ended questions, affirmation skills, making affirmative statements concerning what the person has communicated, the skill of reflecting on the content communicated, and the skill of extracting the essential content, i.e., summarizing what has been said.

1. **Opening of Interview (Engage)**
   - Warm, friendly greeting with a smile
   - Presentation
   - The role
   - Time
   - Establishing an agenda (sketching circles)
   - Request permission

   Example: *Hello, please sit down. My name is _________. How are you today? You are here because your current level of physical activity could be better. Do you agree to talk about it a bit? Tell me a little more about your physical activity.*

2. **Negotiating the work plan (Focusing)**
   - Show the person a drawing with circles
   - Read what is in each circle: *“These topics contain some topics that we can discuss today. These include (...) »*
   - Emphasize the choice: *"Which of these topics would you like to discuss today?"*
   - Encourage collaboration: *"How did you come to choose exactly ...?"*

3. **Ask open-ended questions and encourage conversation about change (Encouragement)**
   - *Does something, and what, concern you about ________?*
   - *If you made a change in your life about this, what benefits would you gain for yourself?*

Use 11 strategies for encouraging change:

A motivational interview helps people go through the natural process of overcoming and addressing ambivalent attitudes they have toward changes in their lives. Remember that the relationship between time spent talking about sustain talk and changing talk represents the best predictor that change will happen at all. Below are 11 approaches you can use to encourage or emphasize the conversation about change. Those are:

1. Ask open-ended questions that encourage conversation
The most direct way to encourage an interviewee to talk about change is to ask him or her directly about it. Ask for: Desire, Ability, Reasons, Confidence, and Need. For example:
«Why do you want to change your current habits regarding ...? Why would you change? What would you achieve with yourself, what is there for you? What opportunities do you see? Why do you want a change? »

2. Determine the importance of change for a person ("Importance ruler")
«How important is it for you to make a change about ..., on a scale of 1 to 10 (0 is completely irrelevant, and 10 is very important)? (below) You rated it 4, why not 0, for example? »

3. Ask questions about balance in decision making
a) Ask what benefit a person has from their present lifestyle, i.e., behavior
b) Ask questions about the disadvantages of the current behavior
c) Ask questions about the disadvantages of the desired behavior
d) Ask what benefits a person can gain from adopting the desired behavior

This order is the best in which you should ask questions regarding decision making. Start by considering the shortcomings of your current behavior or lifestyle, and end by highlighting the benefits a person can have after adopting the desired behavior or lifestyle.

4. Ask for a person to elaborate and explain to you what they mentioned
Ask open-ended questions that shed light on the answers above and help the person gain greater insight and understanding of their own reasons and motives for their current or desired lifestyle. For example:

"In what way (...)? And what else? Can you give an example? Can you tell me a little bit more about the last time it happened to you? How do you think it could be improved? Can you tell me more about that?

5. Continue to encourage the person by reflecting and summarizing what you have heard from them. When you hear a person talk about a change, remember what they mentioned so you can summarize and repeat it in a nice, concise version.

6. Ask the person about the extreme situations of the change and how they affect
«What worries you the most? In your opinion, what would be the worst or best outcome/ outcome if you did not make (or introduce) a change? What do you hope for? If the change was successful, what would be different? »

7. Ask the person to "look back"
«Tell me more about a time when there was no problem. What did you like then? How did it affect you as a person? »

8. Ask the person to “look ahead”
«What do you hope will be different in the future? What would it be like in five years if you did not make any change now? Now imagine that you were quite able to bring about a change, 100% ... what would be different in that case? How would you like your life to look like in five years?”

9. Explore your interviewee's goals and values
«What is important to you in life? What are you dreaming about? What would you like to achieve if you could decide? How does your current behavior affect it? »

10. Ask a person and research what people in the environment think about their current status and change
«Is anyone else in your area interested in making a change with your current lifestyle? Why is this person concerned about your current lifestyle? »

11. «Devil's Advocate»
"Maybe that person is so important to you that you go into the process of making change at any cost ..."

4. Summarize what you have heard

5. Ask for the following steps ("Try Water")

- «What is next? »

6. Close the conversation

- Show that you appreciate coming to the interview: "Thank you!"
- Express that you believe that a person can make a change in their life if they wish: "I am convinced that if you decide to change that part in your life, you will find a way to do it."

+ Non-verbal Body Language

- Establishes and maintains—eye contact
- Facial expression is pleasant, open
- Body posture is open, forward
- Voice tone is warm, pleasant
- Rate of speech is not too slow or too fast
- Spatial proximity is not too close or too distant
